# A patient-derived cell model for malignant transformation in IDH-mutant glioma

**DOI:** 10.1186/s40478-024-01860-6

**Published:** 2024-09-10

**Authors:** Olga Kim, Zach Sergi, Guangyang Yu, Kazutoshi Yamamoto, Martha Quezado, Zied Abdullaev, Danel R. Crooks, Shun Kishimoto, Qi Li, Peng Lu, Burchelle Blackman, Thorkell Andresson, Xiaolin Wu, Bao Tran, Jun S. Wei, Wei Zhang, Meili Zhang, Hua Song, Javed Khan, Murali C. Krishna, Jeffrey R. Brender, Jing Wu

**Affiliations:** 1 Neuro-Oncology Branch, Center for Cancer Research, National Cancer Institute, National Institutes of Health, Building 37, Room 1142A, 37 Convent Drive, Bethesda, MD 20892 USA; 2 Radiation Biology Branch, Center for Cancer Research, National Cancer Institute, National Institutes of Health, Bethesda, MD 20892 USA; 3 Laboratory of Pathology, Center for Cancer Research, National Cancer Institute, National Institutes of Health, Bethesda, MD 20892 USA; 4 Urologic Oncology Branch, Center for Cancer Research, National Cancer Institute, National Institutes of Health, Bethesda, MD 20892 USA; 5 Chemistry and Synthesis Center, National Heart, Lung, and Blood Institute, Rockville, MD 20850 USA; 6 Protein Characterization Laboratory, Leidos Biomedical Inc / Frederick National Laboratory for Cancer Research, Frederick, MD 21701 USA; 7 Genomics Technology Laboratory, Cancer Research Technology Program, Frederick National Laboratory for Cancer Research, Frederick, MD 21701 USA; 8 Sequencing Facility, Leidos Biomedical Inc / Frederick National Laboratory for Cancer Research, Frederick, MD 21701 USA; 9 Genetics Branch, Center for Cancer Research, National Cancer Institute, National Institutes of Health, Bethesda, MD 20892 USA

**Keywords:** Malignant transformation (MT), IDH-mutant glioma, Low-grade glioma (LGG), High-grade glioma (HGG), Tumor mutational burden (TMB), Hypermutator phenotype (HMP), 3D cell model, Matched patient-derived cells

## Abstract

Malignant transformation (MT) is commonly seen in IDH-mutant gliomas. There has been a growing research interest in revealing its underlying mechanisms and intervening prior to MT at the early stages of the transforming process. Here we established a unique pair of matched 3D cell models: 403L, derived from a low-grade glioma (LGG), and 403H, derived from a high-grade glioma (HGG), by utilizing IDH-mutant astrocytoma samples from the same patient when the tumor was diagnosed as WHO grade 2 (tumor mutational burden (TMB) of 3.96/Mb) and later as grade 4 (TMB of 70.07/Mb), respectively. Both cell models were authenticated to a patient’s sample retaining endogenous expression of IDH1 R132H. DNA methylation profiles of the parental tumors referred to LGG and HGG IDH-mutant glioma clusters. The immunopositivity of SOX2, NESTIN, GFAP, OLIG2, and beta 3-Tubulin suggested the multilineage potential of both models. 403H was more prompt to cell invasion and developed infiltrative HGG in vivo. The differentially expressed genes (DEGs) from the RNA sequencing analysis revealed the tumor invasion and aggressiveness related genes exclusively upregulated in the 403H model. Pathway analysis showcased an enrichment of genes associated with epithelial-mesenchymal transition (EMT) and Notch signaling pathways in 403H and 403L, respectively. Mass spectrometry-based targeted metabolomics and hyperpolarized (HP) 1-^13^C pyruvate in-cell NMR analyses demonstrated significant alterations in the TCA cycle and fatty acid metabolism. Citrate, glutamine, and 2-HG levels were significantly higher in 403H. To our knowledge, this is the first report describing the development of a matched pair of 3D patient-derived cell models representative of MT and temozolomide (TMZ)-induced hypermutator phenotype (HMP) in IDH-mutant glioma, providing insights into genetic and metabolic changes during MT/HMP. This novel in vitro model allows further investigation of the mechanisms of MT at the cellular level.

**Graphic Abstract:**

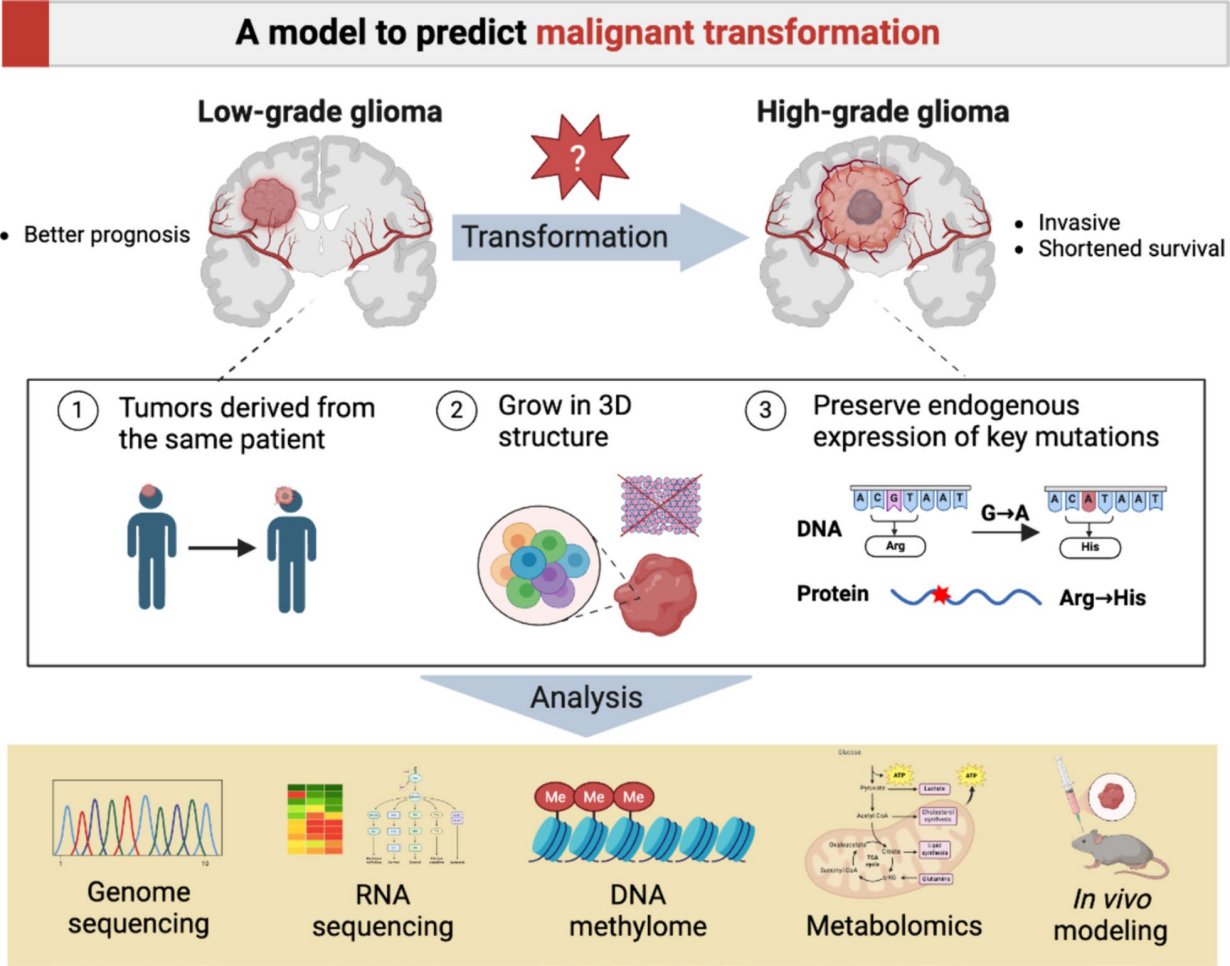

**Supplementary Information:**

The online version contains supplementary material available at 10.1186/s40478-024-01860-6.

## Introduction

Diffuse low-grade gliomas (LGG) harboring *IDH1* R132H mutations are primary brain tumors most commonly diagnosed in patients under 50 years of age [1]. The World Health Organization (WHO) Classification of Tumors of the Central Nervous System (CNS) classifies IDH-mutant gliomas based on histological and molecular features. These tumors can be grade 2, 3, or 4 astrocytomas with intact 1p and 19q chromosomal arms, or grade 2 or 3 oligodendrogliomas with chromosome 1p and 19q codeletion [2]. While IDH-mutant gliomas have a more favorable prognosis compared to IDH-wildtype (IDH-wt), they remain incurable. Additionally, a small subset of progressed tumors accumulates significantly more mutations compared to their parental tumors and develop hypermutator phenotype (HMP) [3]. Most grade 2 or 3 patients undergo disease progression to high-grade (grade 4) tumors over time through a process known as malignant transformation (MT). MT significantly reduces overall survival (OS), with patients experiencing a much poorer prognosis after transformation [4]. Although MT is commonly observed clinically, its mechanisms are not completely understood. Furthermore, non-invasive detection of the MT process remains challenging, hindering timely detection and intervention before the late stage of the process when patients are clinically deteriorating.

Magnetic resonance imaging (MRI) has been associated with less than 80% accuracy in identifying MT; therefore, molecular and pathologic analyses have been applied to define MT [5–7]. There has been an increasing interest in studying MT in IDH-mutant gliomas. A recent retrospective study examined MT in 193 IDH-mutant LGG patients based on pathologic definition of MT by an increase in tumor grade between initial and subsequent surgeries. The study revealed that time to MT is longer in grade 2 oligodendroglioma than grade 2 astrocytoma, and initial gross total resection is associated with delayed MT and improved OS in astrocytoma [4, 8, 9]. Chemotherapy and/or radiation was found to delay MT and prolong MT-free survival in grade 2 oligodendroglioma but may contribute to accelerated MT in grade 2 astrocytoma [4, 10, 11], suggesting a subtype-specific role of treatment in relation to MT and survival. Importantly, a proportion of patients previously treated with temozolomide (TMZ) undergo MT with the development of a HMP characterized by a specific mutational signature [12] and linked to a poor outcome [13], indicating that TMZ-induced HMP is an important prognostic factor and feature of MT.

To gain a better understanding of the genomic landscape dynamics and molecular mechanisms of MT, Bai et al. analyzed fixed tumor samples, comparing higher-grade, progressed samples to their lower-grade IDH-mutant counterparts. Activation of the MYC and RTK-RAS-PI3K pathways and upregulation of the FOXM1 and E2F2 networks and EZH2 were among the oncogenic pathways driving disease progression [14]. However, a pairwise rather than bulk group comparison of the matched samples would be desirable for dissecting case-specific alterations. Jones et al. attempted to model MT and HMP in vitro by establishing IDH1-mutant astrocytoma and IDH1-mutant oligodendroglioma patient-derived cells obtained from surgical tissues of patients at recurrence with chemotherapy-induced hypermutation [15]. However, only tumor tissues at recurrence were characterized and lacked matched tumor samples before MT. Developing experimental models that include matched low-grade and high-grade counterparts that faithfully recapitulate the disease evolution is indispensable in advancing the understanding of the MT process.

Metabolic reprogramming has been recognized as one of the cancer hallmarks required for tumor development and MT in parallel with changes in the genomic landscape [16]. Different metabolic profiles have been reported in different types of gliomas as well as gliomas of different grades, reflecting a complex and dynamic metabolic reprogramming during MT [17, 18]. Unbiased metabolomics flux experiments using a panel of isogenic cell lines containing heterozygous *IDH1/2* mutations revealed elevated glucose turnover in IDH-wt glioma cells and increased dependence on mitochondrial oxidative phosphorylation (OxPhos) in IDH-mutant cells [19]. Yu et al. found that short-chain acylcarnitines and N-methyl-glutamic acid were elevated, whereas lysophosphatidylethanolamines (LPEs), phosphatidylserines (PSs), and phosphatidylinositols (PIs) were decreased in high-grade gliomas compared with grade 2 glioma tissues and para-tumor tissues [17]. However, metabolic shifts specific to *IDH1* mutation associated with MT remain poorly characterized, particularly in the context of the same patient.

Therefore, in this study, we aimed to develop preclinical 3D cell models derived from the same patient before and after MT to facilitate the investigation of the mechanisms of MT and HMP in IDH-mutant glioma at the genetic and epigenetic levels. Importantly, the established cell models are a powerful tool for exploring the key components of metabolic reprogramming at the cellular level, highlighting the advantages of versatility and replicability over single-use fixed tumor tissues.

## Materials and methods

### Generation of patient-derived 3D cell line models

Fresh surgical specimens were obtained from the patient undergoing surgical resection of the tumor at the Surgical Neurology Branch (SNB) at National Institutes of Health (NIH). 403L tumor tissue sample was obtained when the patient’s tumor was diagnosed as WHO grade 2, while 403H sample was obtained from the same patient when the tumor transformed to WHO grade 4.

403L and 403H cell lines were cultured from the 403L and 403H tumor tissues, respectively. The cell lines were generated by the Neuro-Oncology Branch laboratory at the National Cancer Institute (NCI), NIH. Tumor tissues were chopped into small pieces and non-enzymatically dissociated. Both cell lines were grown as 3D tumor spheroids in the NBE medium, composed of Neurobasal -A medium (Thermo Fisher Scientific, Waltham, MA), N2 and B27 supplements (Invitrogen, Carlsbad, CA) and recombinant human basic fibroblast growth factor (bFGF), epidermal growth factor (EGF), and 1 mmol/L of L-Glutamine (Thermo Fisher Scientific). Accutase (Sigma-Aldrich, St. Louis, MO) was used to dissociate 3D tumor spheroids into single cells. GSC923 cells were generated from a tumor tissue of a glioblastoma patient, as described previously [20].

All cells were cultured at 37 °C in 5% CO_2_ and routinely checked for Mycoplasma contamination using MycoAlert Mycoplasma kit (#LT07-318, Lonza).

### Cell line authentication

Cell line authentication was performed using short tandem repeat (STR) profiling. DNAs from cell lines were prepared from 2 × 10^6^ cell pellets for each cell line using DNeasy Blood & Tissue Kit (Qiagen, Germantown, MD), according to manufacturer’s instructions. Germline DNA was extracted from the patient’s blood and served as a reference sample. Each cell line’s STR profile was compared to the patient’s reference sample and the percent match was calculated by adding together the total number of alleles that were identical between the cell line and reference profiles and dividing that number by the total number of total alleles in the cell line profile only; homozygous (one peak at that site) alleles were counted as one. Sample was considered “authenticated”, if the sample had a percent match ≥ 80% when compared to the reference STR profile.

### Droplet digital PCR (ddPCR) mutation detection assay

ddPCR mutation detection assay was used to detect *IDH1* p.R132H mutation in the cell lines. DNA was extracted using DNeasy Blood & Tissue Kit (Qiagen), according to manufacturer’s instructions. The IDH1 p.R132H c.395_396inv probe was purchased from Bio-Rad (Bio-Rad Laboratories, Hercules, CA).

### 3D tumor spheroid invasion assay

The cells were seeded at 5 × 10^3^ cells/well in ultra-low attachment 96-well plates and allowed to naturally form 3D tumor spheroids for 3 days. After confirming formation of one single tumor spheroid in each well, 100 µL of Matrigel (Corning, NY) was gently dispensed per well. The 3D tumor spheroid invasion was monitored using Celigo image cytometer (Nexcelom Bioscience, Lawrence, MA), as described previously [21].

### Western blotting

Tumor spheroids were lysed using RIPA buffer, and the protein concentration of the tumor spheroids was measured using DC (detergent compatible) protein assay. Western blot analysis was performed as described previously [22]. Briefly, protein lysates were resolved on gradient 4–20% gels (Bio-rad) transferred to nitrocellulose membranes and blocked with 5% bovine serum albumin (BSA) in TBS with 0.1% Tween 20 (TBST) for 1 h. Membranes were probed with primary antibodies overnight at 1:5000 dilution for ACTIN and 1:1000 dilution for the rest antibodies used. The next day, membranes were washed with TBST and then probed with secondary antibodies (1:5000 dilution) for 1 h at room temperature. Membranes were visualized by adding Clarity Max Western ECL Substrate (Bio-Rad) and imaged via ChemiDoc Touch imaging system (Bio-Rad). The following antibodies were used: IDH1 R132H (Millipore Sigma, clone HMab-1, #MABC171), SOX2 (Cell Signaling, #3579), GFAP (Cell Signaling, #3670), OLIG2 (Abcam, #ab109186), NESTIN (Millipore Sigma, #MAB5326), beta 3-Tubulin (Cell Signaling, #5568), LDHA (Cell Signaling, #3582), LDHB (Santa-Cruz Biotechnology, sc-100775), and ACTIN (Cell Signaling, #4970).

### Immunofluorescent analysis

Glass slides with removable silicone chambers (ibidi) were pre-coated with Geltrex one hour before cell seeding. The next day after seeding, the NBE culture media was changed to NBE media + 1% FBS to induce differentiation. After 3 days, the cells were washed with PBS and fixed using 4% PFA in PBS for 20 min at room temperature. Following permeabilization using Triton X-100, the cells were incubated with the following primary antibodies: GFAP (Cell Signaling, #3670, 1:1000), beta 3-Tubulin (Cell Signaling, #5568, 1:250), and O4 ((Millipore Sigma, #MAB345M, 1:500). The next day, anti-mouse Alexa Fluor™ 488 (Invitrogen, A-11029, 1:1000) and anti-rabbit Alexa Fluor™ 594 (Invitrogen, A-11012, 1:1000) secondary antibodies were added, and the slides were mounted using DAPI Fluoroshield (Sigma).

### Orthotopic xenograft mouse glioma model using 403L/H cell lines

Eight-week-old female NSG mice were anesthetized with a combination of xylazine (20 mg/ml) and ketamine (100 mg/ml) diluted in 0.9% injection NaCl at a 1:1:4 volume ratios for a total dose of 0.1 ml per 20 g body weight. After the animals were fully anesthetized, they were placed and immobilized in a small animal stereotactic frame fitted with a mouse-specific headpiece using lidocaine gel pre-treated ear bars. All surgery procedures were done under sterile condition. 403L and 403H cells (1 × 10^6^ cells/4µL) resuspended in serum-free Neurobasal -A medium were slowly injected intracranially (2 mm anterior and 2 mm lateral to bregma; 2.5 mm deep from the dura). The burr hole was closed with bone wax, and the scalp closed with Vetbond, or one to two staples or sutures. Five mice were injected with each cell line. Animals were observed frequently while they recover from the anesthesia in a warm, draft-free area. All animal experiments were approved by the National Cancer Institute Animal Care and Use Committee (NCI ACUC) and conducted in accordance with NCI ACUC guidelines under the authority of the animal protocol (NOB-023). Animal behavior and body weight were monitored constantly. Animals were euthanized when they reached the endpoints. The experimental endpoint criteria for intracranial tumors include symptoms of increased intracranial pressure such as less moving around the cage and appearing drowsy with less response to stimuli; signs of neurological impairment such as paralysis, circling, head tilting and conclusion; swollen/domed cranium; swelling subcutaneously at injection site > 1 cm diameter or that adversely affects the general well-being of the mouse. Additional humane endpoint criteria include body weight loss (> 15%), rough hair coat, hunched posture, rapid or labored breathing, debilitating diarrhea, dehydration, lethargy, jaundice, pallor or cyanosis, anemia, bleeding from any orifice, self-induced trauma, impaired mobility affecting the ability to reach food or water.

### Magnetic resonance imaging (MRI)

Mice were anesthetized prior to MRI imaging with isoflurane inhalation (4% for induction and 1%–2% for maintenance) and placed in a prone position. Axial and coronal images were acquired using a Rapid Acquisition with Relaxation Enhancement (RARE) sequence on a Bruker BioSpec 3T scanner. The acquisition parameters included a matrix size of 128 × 128, a field of view of 20 mm × 20 mm, a slice thickness of 0.5 mm, a repetition time of 2367 ms, and an echo time of 48 ms. Throughout the MRI procedure, the mice's breathing rate was monitored with a pressure transducer (SA Instruments, Inc.) and maintained at 60 ± 20 breaths per minute. The core body temperature was kept at 36 °C ± 1 °C using warm air flow. MRI procedures were approved by the NCI ACUC and in accordance with federal regulatory requirements and standards.

### Histology and immunohistochemistry (IHC)

Patient’s surgical specimens and whole brains from the mouse xenograft models containing non-tumor and tumor regions were fixed in 10% formalin, embedded in paraffin, sectioned at 4 μm, and mounted on slides. Hematoxylin and eosin (H&E) staining was performed per standard protocol. All slides were stained on the Ventana Benchmark Ultra automated immunostainer using a standard DAB protocol. The following antibodies were used for the IHC analysis: IDH1 R132H (Dianova, clone H09, DIA-H09; 1:100), ATRX (Sigma, #HPA001906, 1:200), and Ki-67 (Dako, clone MIB-1, M7240; 1:200).

### Whole exome sequencing (WES) and tumor mutational burden (TMB) of tumors

Whole exome sequencing (WES) was carried out on paired tumor and germline DNA, which was extracted from formalin fixed paraffin embedded (FFPE) tumor tissue and peripheral blood mononuclear cells (PBMC), respectively. Exome libraries were prepared using Agilent SureSelect Clinical Research Exome kits (Agilent, Santa Clara, CA) and sequenced using paired-end method on Illumina NextSeq500 sequencers (Illumina, San Diego, CA). Germline and somatic mutations were called as described previously [23]. Tumor mutational burden (TMB) was calculated as number of somatic mutations per megabase (Mb) of sequenced genomic region. The somatic mutations variants tier system was used as it was detailed previously [24].

### DNA methylation analysis of tumors and cell lines

Genomic DNA (250 ng as the standard) was extracted from FFPE tissue sections using the AllPrep DNA/RNA FFPE kit (Qiagen, Hilden, Germany) and bisulfite-converted (EZ DNA Methylation Kit, Zymo Research D5001). Bisulfite-converted FFPE DNA was processed with the Infinium FFPE DNA Restore kit (Illumina) and assayed on Infinium MethylationEPIC kit (Illumina), according to the Infinium HD FFPE Methylation Assay automated protocol (Illumina). Methylation data was processed using versions 11.b4 and 12.5 of the DKFZ classifiers and NCI-Bethesda classifier [25]. For clustering and dimensionality reduction, feature selection was performed by reducing the beta value matrix to the most variable probes by standard deviation (> 0.21). The number of principal components (PC) used for dimensionality reduction were 40. Dimensionality reduction on the PC matrix was then performed using the uniform manifold approximation and projection (UMAP) method (uwot R package) with the following non-default parameters: n_neighbors = 10, spread = 2, min_dist = 0.2.

Genomic DNA from cell models was prepared from 2 × 10^6^ cell pellets. DNA was extracted using DNeasy Blood & Tissue Kit (Qiagen), according to manufacturer’s instructions. EPIC 850 k array was used for methylation profiling of the samples. The array data was run through the ChAMP package’s EPIC pipeline resulting in a matrix of normalized beta values for further analysis [26]. The CpG sites of interest were categorized into four groups: Both Methylated (Beta > 0.75 in both cell lines), Both Unmethylated (Beta < 0.25 in both cell lines), 403H Methylated Only, and 403L Methylated Only [26]. CpG sites were mapped to CpG islands and gene regions using the publicly available EPIC manifest file.

### RNA sequencing and Gene set enrichment analysis (GSEA) of cell models

RNA from cell models was extracted using PureLink RNA Mini Kit (#12183018A, Thermo Fisher Scientific) following manufacturer’s instructions, including on-column DNase treatment to remove contaminating DNA (#12185010, PureLink DNase Set, Invitrogen). First, raw RNA-Seq data was filtered via the filter by expression function from the Edger package. Next, the counts were normalized and statistically compared with the DESeq, results (lfcThreshold = 0.58, alpha = 0.05), and lfcShrink functions from the DESeq2 package. Pathway enrichment analysis was performed using Gene Set Enrichment Analysis (GSEA) on DESeq2 rlog transformed counts with the Signal2Noise ranking metric. The Hallmark collection within the Molecular Signatures Database (MSigDB) was analyzed for enrichment with the GSEA algorithm. Only enriched gene sets with an FDR value less than 0.05 were considered for further analysis.

### Liquid chromatography–mass spectrometry (LC–MS) measurements of cell metabolites

Cells were seeded at 2 × 10^6^ cells in T25 flasks in two cell culture media conditions: (1) regular NBE medium and (2) NBE medium without L-glutamine. The cells were cultured in suspension as 3D tumor spheroids for 3 days and four replicates were collected for each cell line and growth condition.

Isotope dilution liquid chromatography-mass spectrometry was performed to measure the concentrations of metabolites in several key cellular metabolic pathways. In brief, all reference target compounds, and isotopic standards were purchased from Sigma-Aldrich, Cambridge Isotope Laboratory, Medical Isotopes, Inc. and CDN Isotopes Inc. Cell pellets were extracted by chilled 80% methanol in water supplemented with appropriate isotopic standards. Reversed-phase LC-MS2 analysis was performed using Thermo TSQ™ triple quadrupole mass spectrometers (Thermo Fisher Scientific) coupled to either Shimadzu 20AC-XR or Vanquish (Thermo Fisher Scientific) liquid chromatographic system for the measurements of central carbon metabolites including R and S forms of 2-hydroxyglutaric acids, free fatty acids, acylcarnitines, and cholesterol, respectively. The mass spectrometers were operated in either negative or positive ion mode and set to monitor parent-product ion transitions using Selected Reaction Monitoring (SRM). Quantitation of targeted metabolites was carried out using Xcalibur™ Quan Browser (Thermo Fisher Scientific). Calibration curves for each metabolite were constructed by plotting reference compound/isotopic peak area ratios obtained from the calibration standards curve and fitting the data using linear regression with 1/X weighting. The analyte concentrations in samples were then interpolated using the linear function obtained from the calibration curve.

### Real-time mito stress assay

The cells were seeded into 96-well Seahorse microplate (Agilent, Santa Clara, CA) at 0.04 × 10^6^ cells/well density. Sensory cartridge was hydrated in a non-CO_**2**_, 37 °C incubator for 12–18 h prior to analysis. Next day, the cells were analyzed real-time on a Seahorse XFe96 Analyzer using a Seahorse XF96 Cell Mito Stress Test Kit (Agilent), according to the manufacturer’s instructions. The data were normalized to protein content measured by the DC protein assay (Bio-Rad).

### Hyperpolarized 1-^13^C pyruvate nuclear magnetic resonance (NMR) of cell models

Accutase was applied in cells cultured in 2D (80% confluence), washed twice in high glucose Hans Clever media, and resuspended in high glucose Hans Clever media lacking sodium pyruvate media. A total of 400 μL of the cell solution (2.0 × 10^7^–5.0 × 10^7^ cells per sample) was transferred to a 5-mm NMR tube. This sample was incubated in a 37 °C water bath for 5 min. 150 μL of 94.6 mM hyperpolarized 1-^13^C pyruvate solution was added to the cell mixture, resulting in a final pyruvate concentration of 25.8 mM and pH of 7.5. 150 scans of one dimensional ^13^C NMR spectra were acquired immediately after the addition of the hyperpolarized 1-^13^C pyruvate solution. NMR measurements were conducted on a 1 T Magritek bench top NMR, using a pulse length of 5 degree and repetition time of 2 s.

### Lactate dehydrogenase (LDH) assay

LDH activity was examined in 2 × 10^6^ cell pellets using quantitative LDH enzyme assay kit (Abcam, ab102526), according to manufacturer’s instructions. The data were normalized to the protein content measured by the DC protein assay.

### Statistical analysis

Statistical analyses were performed using GraphPad Prism software (version 9.3.1, San Diego, CA). Orthogonal partial least squares-discriminant analysis was performed on the log transformed, mean centered concentrations normalized to total metabolites using MetaboAnalyst 6.0 [27]. The data is expressed as mean ± SEM. A 2-tailed *t-*test was used to evaluate a difference between experimental groups. *P* < 0.05 was considered statistically significant.

## Results

### Patient’s clinical history and characteristics

**Fig. 1:**
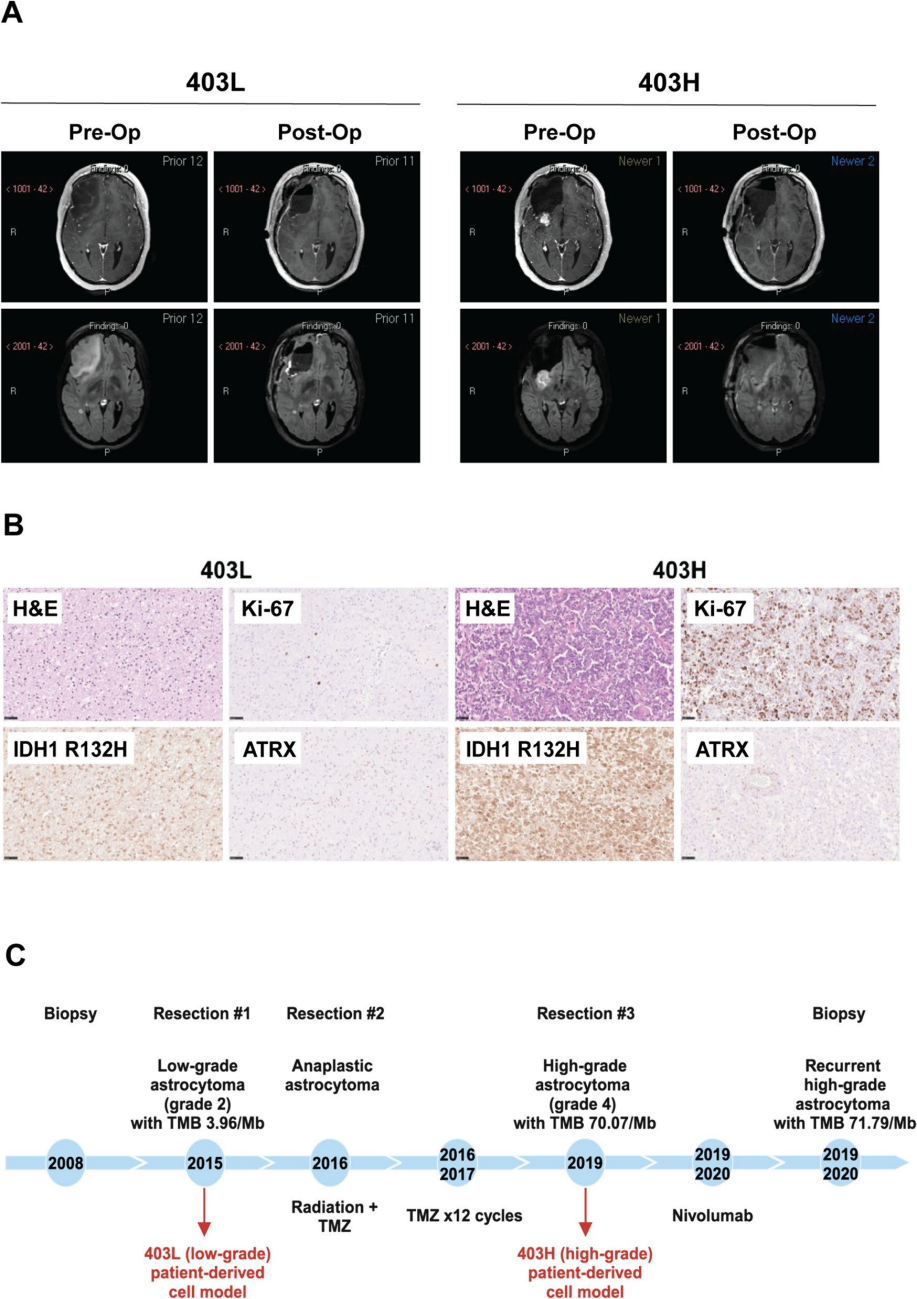
Case presentation. **A** Left panel: patient’s MRI obtained before (pre-op) and after (post-op) resection #1, when the tumor was low-grade astrocytoma (403L). Right panel: The same patient’s MRI obtained before (pre-op) and after (post-op) resection #3, when the tumor was transformed to high-grade astrocytoma (403H). **B** H&E staining showing tumor pathology and IHC analysis demonstrating Ki-67 expression, IDH1 R132H mutation, and ATRX loss (10 × magnification) in 403L and 403H patient’s tissues following 1st and 3rd resections, respectively. **C**. Timeline of disease diagnosis and treatment. Red arrows indicate that 403L and 403H patient-derived cell lines were cultured from the freshly resected tumors at resections #1 and #3, respectively

The patient was a woman in her 30 s with a right frontal astrocytoma, biopsied as WHO grade 2. She was subsequently followed with serial imaging until she became symptomatic, and brain MRI suggested disease progression with a large non-enhancing lesion in the right cerebral hemisphere causing a mass effect with a midline shift (Fig. 1A). She underwent a gross total resection (Fig. 1A), and pathology was notable for LGG with *IDH1* mutation, WHO grade 2 (403L, Fig. 1B). Before she was able to get the tumor treatment, she had a second tumor resection. Results of the WES of tumor tissue were compatible with astrocytoma (*ATRX* loss and *TP53* mutation). She then received concurrent radiation and TMZ followed by twelve cycles of adjuvant TMZ. Unfortunately, tumor progression was observed again, and a third tumor resection was performed eleven years after the initial diagnosis. Pathological examination along with DNA methylation suggested high-grade astrocytoma, IDH-mutant, WHO grade 4 (403H, Fig. 1B). IHC staining detected a strong expression of the IDH1 R132H mutant and loss of ATRX in the nuclei of both 403L and 403H surgical tumors. Ki-67, a proliferative marker, showed a moderate-to-strong expression in the 403H tumor only (Fig. 1B). In addition, WES demonstrated TMB of 3.96/Mb and 70.07/Mb at grades 2 and 4, respectively (Fig. 1C). MT was diagnosed based on the clinical, histopathological, and genomic evaluation of the initial (grade 2) and recurrent (grade 4) surgical tumors. In addition to the TMB and high confidence mutations in tumor samples from all three surgical resections in the reported time frame, WES analysis of 403H revealed mutational signature 11, suggesting TMZ-induced HMP (Suppl. Fig. S1, Table S1) [12].

### Patient-derived cell models express mutant IDH and possess invasive and stem-like properties

As shown in the Fig. 1C, the 403L (low-grade) and 403H (high-grade) patient-derived cell lines were cultured from the freshly resected tumor tissues obtained from the same patient when they were diagnosed as grades 2 and 4, respectively. STR analysis using gDNA obtained from both cell lines confirmed a match to the patient’s germline (100% in 403L and 93.33% in 403H) (Table 1). Additionally, ddPCR using the specific *IDH1* R132H probe detected that the mutation fraction abundance in 403L and 403H cells were 45.56 and 41.92, respectively (Suppl. Fig. S2A). Western blot analysis verified expression of IDH1 R132H in both cells (Suppl. Fig. S2B). These results verified the authentication of the cell models generated from the tumors of the same patient harboring the *IDH1* R132H mutation.

**Table 1:** Short tandem repeat (STR) analysis of cell models versus patient’s sample

STR loci tested	Reference (Patient's sample)	403L cells sample	403H cells sample
D3S1358	14, 15	14, 15	14, 15
D7S820	9, 11	9, 11	9, 11
vWA	15, 17	15, 17	15, 17
FGA	19, 21	19, 21	18, 19, 21
D8S1179	10, 13	10, 13	10, 13
D21S11	27, 32.2	27, 32.2	27, 32.2
D18S51	13, 19	13, 19	13, 18, 19
D5S818	10, 13	10, 13	10, 13
D13S317	11, 12	12	12
D16S539	12, 13	12, 13	12, 13
TH01	9, 9.3	9	9
TPOX	11	11	11
CSF1PO	10, 11	11	10, 11
AMEL	X	X	X
Penta D	9, 13	9, 13	9, 13
Penta E	7, 14	7, 14	7, 14
Mouse	NA	NA	NA

*NA* Allele information not available

No mouse DNA was detected for both cell samples

**Fig. 2:**
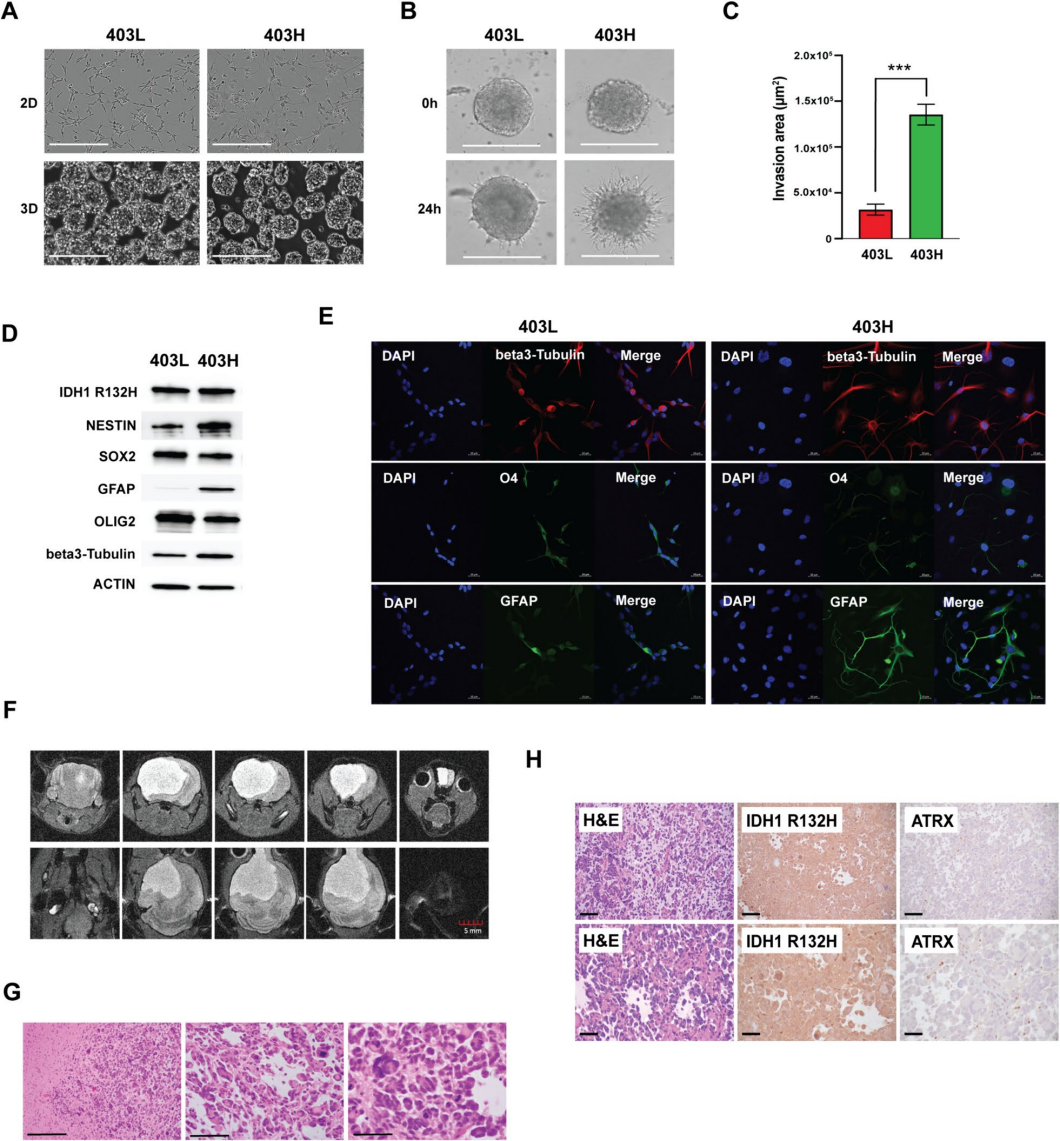
In vitro characterization of the established LGG and HGG models. **A** 403L and 403H cell growth in 2D monolayer and 3D spheroid cultures (scale bar 400 µm). **B** 3D spheroid invasion assay in 403L and 403H measured by Celigo Image Cytometer over 24 h of 3D culture (scale bar 500 µm). **C** Invasion area measured by ImageJ in 403L and 403H at 0 h and 24 h. **D** Western blot analysis of 403L and 403H spheroids showing expression of IDH1 R132H, stem cell and lineage markers. **E** Immunofluorescent analysis of beta3-Tubulin (neuronal), O4 (oligodendroglial), and GFAP (astrocytic) lineage markers in 403L and 403H cells cultured as 2D monolayers in the culture media containing 1% FBS for 3 days. **F** MRI of the 403H orthotopic mouse model suggesting tumor growth. **G** H&E staining of the 403H murine tumors displaying tumor infiltration (left; scale bar 200 µm), polymorphic and bizarre cells (middle; scale bar 100 µm) as well as multiple atypic mitoses (right; scale bar 50 µm). **H** H&E (left), IHC staining of IDH1 R132H (middle) and ATRX (right) in 403H xenografts (top—scale bar 100 µm; bottom—scale bar 50 µm)

As shown in Fig. 2A, the 403L and 403H cell models were able to grow in 2D culture on the pre-coated surface as well as to form 3D tumor spheroids in suspension. As the ability for cell migration and invasion are among the key characteristics of the malignant phenotype, we examined 403L and 403H cell models in 3D tumor spheroid invasion assay. At 24 h, 403H spheroids showed a marked increase in the area invaded by cells around the spheroid compared to 403L (*p* < 0.0001) (Fig. 2B, C). These results indicated that 403H has a higher potential to invade and spread into nearby areas as compared to 403L.

Next, we explored the expression of stem cell markers in 3D spheroids. The levels of stem cell marker SOX2 and neuronal marker beta3-Tubulin were nearly equal. Interestingly, 403H had a higher expression of NESTIN, which is known as a neural stem/progenitor cell marker. We found that both models had OLIG2-expressing cells with higher levels in 403L and GFAP-positive astrocytic cells mostly in 403H (Fig. 2D). In order to explore the multilineage potential, 1% FBS was introduced to the culture medium, as described previously [28]. When both cells were cultured in the media containing FBS to induce differentiation, we observed the expression of all three lineage markers using immunofluorescent staining (Fig. 2E). Overall, these results suggest that the established cell models possess stem cell-like traits and a multilineage potential. Collectively, 403L and 403H cells cultured from the patient’s tumors were established as stable 3D neurospheres retaining mutant IDH and displaying hallmarks of invasiveness, self-renewal, and multipotency.

### 403H develops HGG tumors in an orthotopic mouse xenograft model

Next, we injected 403L and 403H cells intracranially into NSG mice to test their tumor formation capacity in vivo. An MRI scan was performed at 14.5 months post-injection with 403H cells and demonstrated a large area of T2 hyperintensity in mouse brain suggesting tumor growth (Fig. 2F). Histologically, the 403H tumors were infiltrative HGG tumors (Fig. 2G), demonstrating tumor infiltration (left), polymorphic and bizarre cells (middle) as well as multiple mitotic figures (right). IHC analysis confirmed IDH1 R132H expression and ATRX loss in the nuclei of tumor cells (Fig. 2H). 4 out of 5 mice injected with 403H cells developed tumors with a median survival of 13.5 months. No tumor development was observed in mice injected with 403L cells within 18 months of observation. These results suggest that 403H cells can form aggressive high-grade tumors in brain microenvironment that resembles human HGG. Consistent with previous reports, it remains challenging to establish a low-grade IDH-mutant orthotopic glioma mouse model [15, 29].

### DNA Methylation analysis of LGG and HGG models

**Fig. 3:**
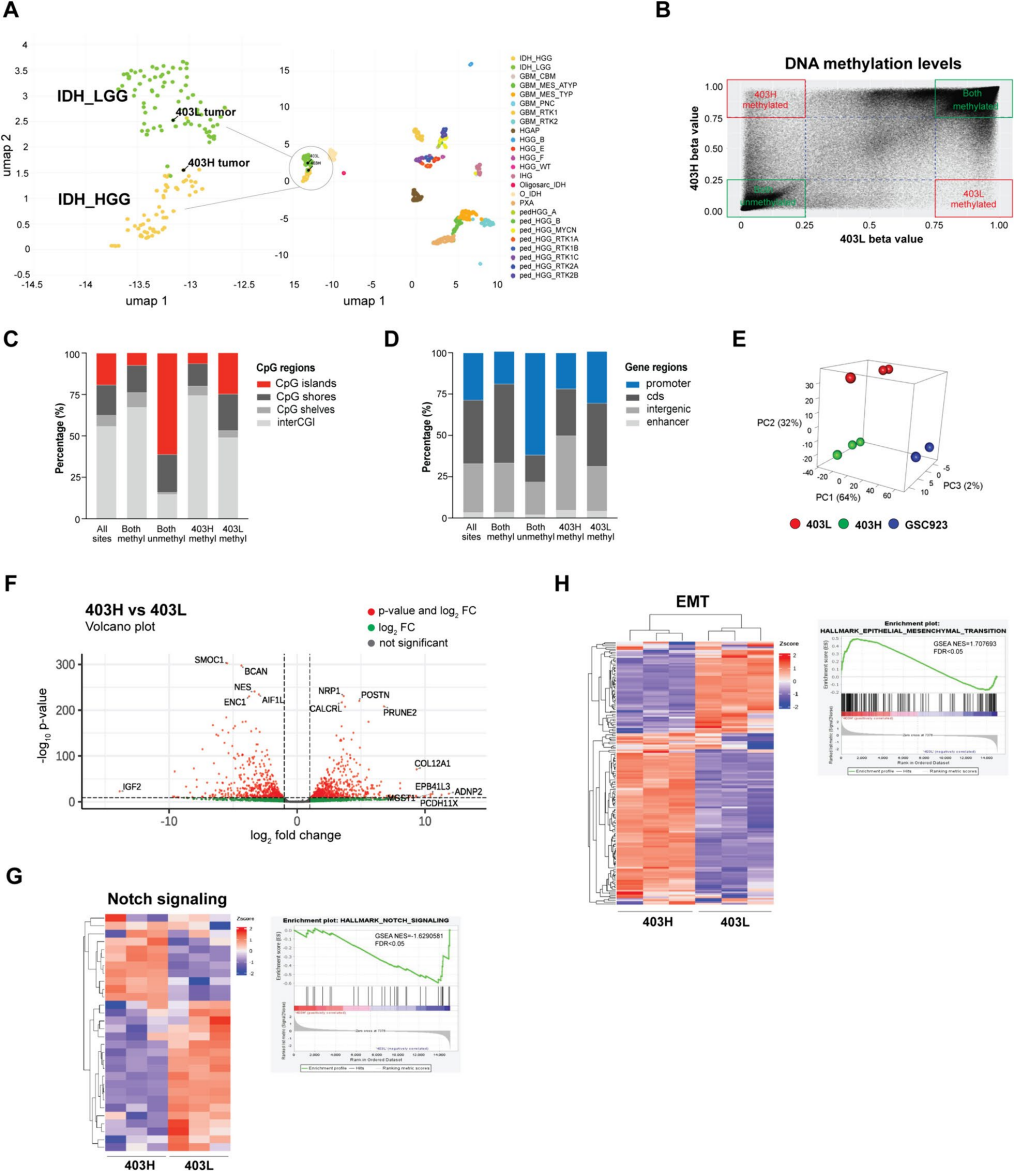
DNA methylation and RNAseq analyses of the LGG and HGG parental tumors and cell models.** A** UMAP demonstrating DNA methylation-based clustering of the parental 403L and 403H surgical tissues. **B** DNA methylation patterns across the genomes of 403L and 403H cells based on normalized beta values. Red and green boxes represent areas of CpG sites methylated in both cell lines or specific to either 403L or 403H. **C** Distribution of CpG regions across grouped CpG sites. **D** Distribution of gene regions across grouped CpG sites. **E** PCA plot comparing RNAseq samples for 403L, 403H, and GSC923 cells. **F** Volcano plot of the DEGs in 403H versus 403L comparison based on *p*-value and log_2_FC. **G** Notch signaling heatmap and enrichment plot in 403L and 403H. **H** EMT signaling heatmap and enrichment plot in 403L and 403H

UMAP analysis was employed to investigate DNA methylation profiling of the tumors using methylation-based classifiers of the CNS tumors. As expected, the parental tumors 403L and 403H were clustered together under the IDH-mutant glioma category in IDH_LGG and IDH_HGG subclasses, respectively (Fig. 3A). Next, we conducted a comprehensive examination of DNA methylation patterns of 742,000 CpG sites across the genomes of the two cell lines, 403L and 403H. To assess the presence of methylation at each CpG site, beta values were employed, as described previously [26]. Notably, we observed that 60% of the CpG sites displayed methylation in both cell lines (top right quadrant in green). Additionally, 18% of the CpG sites showed a consistent absence of methylation in both cell lines (bottom left quadrant in green). Conversely, a subset of CpG sites amounting to < 1% emerged as exclusively methylated in each respective cell line (top left and bottom right quadrants in red) (Fig. 3B). Further analysis of the methylated regions showed an inverse enrichment of CpG islands (CGI) and open sea probes (interCGI) among both methylated and both unmethylated groups (Fig. 3C). Analysis of gene regulatory regions similarly showed inverse patterns of methylation in coding sequence (cds) and in promoter regions (Fig. 3D). The 403L methylated sites showed an enrichment for CGIs and promoter regions compared to 403H methylated sites (Fig. 3C, D). Overall, the high percentage of consistency in DNA methylation profiles between the established cell lines support their identity and a common cell of origin.

### Notch signaling and epithelial-mesenchymal transition (EMT) are important during MT

To gain insight into the underlying biological distinctions between 403L and 403H at the gene expression level, RNAseq analysis was performed using 403L, 403H, and a patient-derived IDH-wt cell line GSC923. Each cell line showed a distinct separation on the principal component analysis (PCA) plot. The first component PC1, accounting for 64% of the variance, separated the IDH-wt GSC923 cell line from the IDH-mutant cell lines. The second component PC2, accounting for 32% of the variance, distinguished the 403L and 403H cell lines within the IDH mutants (Fig. 3E). Top differentially suppressed genes as the tumor transformed included *SMOC1, BCAN*, *NES, AIF1L, ENC1*, and *IGF2* (*p* < 0.01). In contrast, the expression of *NRP1, POSTN, CALCRL, PRUNE2, COL12A1, ADNP2, EPB41L3, PCDH11X,* and *MGST1* was significantly higher in HGG than LGG model (Fig. 3F, *p*  < 0.01). When we performed a pathway analysis of DEGs using GSEA, Notch signaling and epithelial-mesenchymal transition (EMT) emerged as statistically significant when compared to one another. As shown in heatmaps and enrichment plots in Fig. 3G and H, the 403L cell line showed an enrichment of genes related to the Notch signaling pathway, such as *NOTCH1* and *NOTCH3*, while the 403H cell line exhibited a vast upregulation and enrichment of EMT genes, such as *NRP1, POSTN, CALCRL, COL12A, ADNP2, PCDH11X,* and *MGST1*.

### The cell models provide insight into metabolic reprogramming during MT in IDH-mutant gliomas

**Fig. 4:**
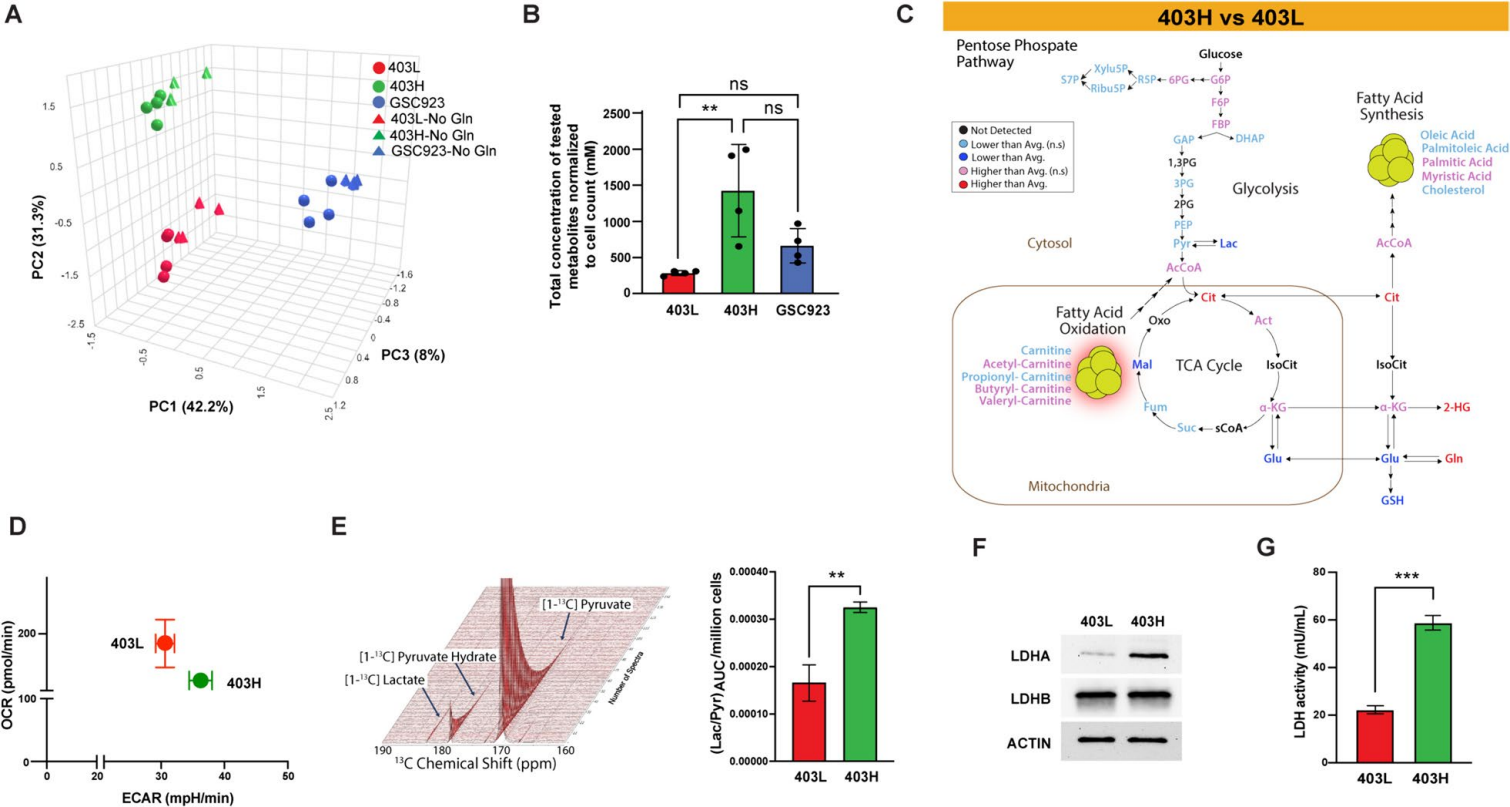
Metabolic characterization of the established models.** A** PCA plot of the three cell cell lines and two media conditions (Blue = GSC923, Red = 403L, Green = 403H, Circles = Media with 1 mM glutamine, Triangles = Media without glutamine). **B** Bar graph showing a total concentration of tested metabolites normalized to cell count in each cell line. **C** Statistically significant metabolites for the 403H vs 403L comparison plotted on the major metabolite pathways. **D** Energy map showing real-time measurement of maximal OCR and ECAR levels in live 403L and 403H cells. **E** Hyperpolarized ^13^C pyruvate NMR spectroscopy measuring Lac/Pyr ratio in 403L and 403H. **F** Western blot of LDHA and LDHB expression in 403L and 403H. **G** LDH activity in both cell lines

Since *IDH* mutation leads to metabolic reprogramming that affects various cellular processes including energy production and redox regulation [30, 31], we utilized LC–MS based metabolites profiling of in vitro 3D cell cultures of 403L, 403H, and GSC923 to identify distinct metabolic signatures associated with *IDH* mutation status, MT, and glutamine dependency. We also explored the effects of glutamine supplementation on the metabolic profiles in the cell lines. PCA of the metabolites revealed that all samples clustered by cell line (Fig. 4A). The first component, accounting for 42.2% of the variance, separated the IDH-wt GSC923 cell line from the IDH-mutant cell lines. The second component, accounting for 31.3% of the variance, distinguished the 403L and 403H cell lines within the IDH mutants. The third minor component, accounting for 8% of the variance, was largely associated with the presence or absence of glutamine in the culture medium. As the contribution of culture medium was minor, we only considered cells cultured in the presence of glutamine in subsequent analysis.

Total metabolite concentrations are shown in Fig. 4B. All tested metabolites, except for succinate and AMP, had a higher absolute concentration in the 403H cell line compared to 403L cells (Suppl. Fig. S3). To adjust for the difference in the metabolic activity and to reveal significantly altered metabolites in each cell line, metabolite levels were normalized to the total sum of the metabolites within each cell line. Compared to the 403L cell line, 403H is characterized by a relative accumulation of intermediates in the first half of the TCA cycle and a relative depletion of TCA cycle intermediates after the second half (Fig. 4C). There was a relative increase in the production of early glycolytic intermediates and decrease in lactate when compared to their levels across all metabolites in the 403H vs 403L. When comparing the normalized metabolite levels between 403H and 403L, fatty acid metabolism, including the synthesis of palmitic and myristic acids and the FAO of acetyl-, butyryl-, and valeryl-carnitines, was relatively more pronounced in 403H. Importantly, glutamine and D-2HG levels were significantly higher in 403H compared to 403L, when normalized to cell count and total sum of metabolites within each cell line (Fig. 4C and Suppl. Fig. S3). In addition, OPLS-DA analysis revealed that the IDH-mutant cell lines are primarily separated from the IDH-wt cell line by elevated 2-HG, an increase in early glycolytic intermediates such as fructose biphosphate and fructose 6-phosphate, as well as decreased fatty acid synthesis (Suppl. Figs. S4 & S5). Overall, the normalized metabolite profiles are consistent with a metabolic shift in the 403H cell line, characterized by a relative decrease in production of late glycolytic intermediates and a corresponding relative upregulation of fatty acid metabolism and glutamine synthesis.

To assess the glycolytic and mitochondrial respiration potential in live 403L and 403H cells, we performed the Seahorse mito stress assay. As shown in Fig. 4D, the maximal respiratory capacity, measured by the oxygen consumption rate (OCR) levels after adding carbonyl cyanide-p-trifluoromethoxyphenylhydrazon (FCCP), was slightly higher in 403L compared to 403H cells, but this difference was not statistically significant (*p* = 0.2182). The extracellular acidification rate (ECAR), which represents glycolytic flux, was significantly higher in 403H cells (*p* = 0.0139). These findings suggest that both IDH-mutant models have comparable levels of OxPhos, but the 403H cells exhibit a slightly higher glycolytic capacity (Fig. 4D).

To better understand the metabolic flux through lactate dehydrogenase (LDH) and to complement our previous measurements of steady-state lactate concentrations, we performed hyperpolarized 1-^13^C pyruvate in-cell NMR spectroscopy on 403L and 403H cell models. This technique allows for the real-time observation of metabolic flux, providing insights into the dynamic processes that steady-state measurements cannot capture as they depend on the activity of multiple pathways. Hyperpolarized measurements, by contrast, are a transient phenomenon that measures the metabolic conversion through a single reaction with the product to substrate ratio being proportional to the metabolic flux through that reaction. The analysis demonstrated that 403H cells had a higher lactate/pyruvate (Lac/Pyr) ratio compared to 403L, with mean (Lac/Pyr)AUC/million cells being 1.667 × 10^–4^ and 3.251 × 10^–4^ in 403L and 403H cells, respectively (*p* < 0.01) (Fig. 4E).

To further investigate the mechanisms behind these differences, we examined lactate dehydrogenase A (LDHA) and B (LDHB) expression levels and LDH activity in both cell models. Our analysis revealed that while LDHB levels were similar between the two models, LDHA expression was significantly higher in 403H cells (Fig. 4F). A quantitative LDH assay confirmed increased LDH activity in the 403H model (Fig. 4G), supporting the elevated Lac/Pyr ratio observed in the hyperpolarized 1-^13^C pyruvate measurements (Fig. 4E). These results suggest enhanced glycolytic capacity in 403H cells, driven by higher LDHA expression and LDH activity, compared to 403L cells. In summary, analysis of metabolic profiles of the established 403L and 403H cells revealed alterations in the fatty acid metabolism, TCA cycle, glutamine synthesis, and glycolysis in MT similar to those associated with the *IDH* mutation [32].

## Discussion

Despite an overall better prognosis in IDH-mutant gliomas, which often present as LGGs, most tumors recur, leading to relentless MT and hypermutation in a subset of transformed tumors. The development of preclinical models that closely resemble tumor biology changes during MT as well as HMP induced by TMZ in IDH-mutant glioma are crucial in advancing understanding of the underlying mechanisms and discovering new targets and factors of the disease. Preclinical models are also of great importance as a tool to test the efficacy of novel therapeutics and discovering causes of drug response or resistance. For the first time, we developed a preclinical model of MT with TMZ-induced HMP by establishing matched patient-derived 3D cell lines of LGG and HGG that were established from the same patient at grades 2 and 4, respectively. Both models recapitulated the main characteristics of the parental tumors at genetic and epigenetic levels. HGG model demonstrated a superior capacity for cell invasion evidenced in 3D invasion assay and upregulation of the EMT pathway in the RNAseq analysis. Importantly, the cell models allow the investigation of the metabolites at the cellular level, which can be challenging in the in vivo setting. The LC–MS based metabolite profiling revealed alterations in TCA cycle, glycolysis, glutamine and fatty acid metabolism and observed an overall higher metabolic activity in the HGG model compared to LGG. Utilizing hyperpolarized 1-^13^C pyruvate in-cell NMR spectroscopy, we were able to detect the metabolic flux suggesting an increasing lactate/pyruvate ratio when the MT occurs in the cell model. To our knowledge, this is the first study reporting the development of the matched patient-derived 3D models of MT with HMP, offering new insights into better understanding of the MT process in IDH-mutant glioma. This novel in vitro 3D model presents opportunities to be used as a tool for further elucidation of the mechanisms of MT.

Developing models of MT in IDH-mutant glioma has been challenging due to various reasons. Firstly, HGG models are easier to develop because of the fast-growing nature. In contrast, it has proved to be challenging to establish a truly LGG glioma cell lines from patients’ tissues. Verheul et al. have attempted to establish cell cultures from over 275 LGGs and HGGs, of which only 12 cultures were successfully established. All the 12 cell lines appear to represent a more advanced stages of astrocytomas, even when derived from LGG tumors [29]. Secondly, since the development of patient-derived cell lines with endogenous mutations has been a challenge, introducing *IDH* mutation and other genetic alterations to the IDH-wt HGG models has frequently been used as an approach to investigate associations between IDH-mutant and IDH-wt tumors as well as LGG and HGG [33, 34]. However, the intrinsic genetic background of IDH-wt HGG tumors, which harbor abnormalities rarely found in LGG tumors, may lead to a distorted interpretation of the findings. Thirdly, since the timing of MT is unpredictable, it is challenging to capture the opportunity for tumor sample collection from the same patients to make the ideal paired comparisons. Among prominent advantages of the 3D models established in this study are that they were derived from the same patient before and after MT, express endogenous *IDH* mutation and share genetic and epigenetic similarities with their parental tumors. Additionally, it is important to highlight the ability of our cell models to grow in 3D spheroids, conferring specific advantages over 2D models. Since 3D spheroids grow at a slower rate than 2D cell culture that grows at an unnaturally rapid pace, this makes 3D spheroids more suitable for testing long-term effects of drugs since they can remain stable for a prolonged period. Moreover, 3D spheroids can replicate more natural tumor architecture by comprising of an external proliferating zone, an internal quiescent zone with limited oxygen, nutrient and growth factor distribution, and a necrotic and hypoxic core [35]. Importantly, all these aspects affect drug response and make 3D spheroids extremely useful in drug discovery.

Conversely, one of the limitations of this paired model and other tumor models is that since they are comprised of the tumor cells, they represent only a monotypic tumor cell population that lacks tumor microenvironment (TME). To model TME and enhance the complexity of such models, the cells could be co-cultured with stromal cells, such as immune cells, endothelial cells, and fibroblasts. Such approach may lead to a closer resemblance to the real tissue and offer new insights into the impact of non-tumor cell types on MT process and drug response. Additionally, as the tumor developed HMP, the biological, genomic, and metabolic changes between our cell line models may not reflect those in MT without HMP.

Our observation that only HGG model was able to grow tumors in vivo, when the cell cultures were injected intracranially points to the aggressiveness of the HGG model which can grow and expand effectively in a xenograft model. The challenge of developing LGG models that grow tumors in vivo is in line with the previous study where researchers established 12 IDH-mutant cell cultures derived from patients’ tumors, yet none of them formed tumors in vivo [29]. However, it was reported previously that even if xenografted human cancer cells grow in vivo, the paracrine interaction of xenografted human cancer cells with the surrounding mouse tissues may not be complete and highly tumor-stromal relevant cross talk may be missing [36, 37]. These are some of the aspects of the cross-species incompatibility observed in xenograft models that ultimately points to a consideration that different methodologies should be used to complement each other for a rigorous research and preclinical testing.

Pathway analysis of the RNAseq data helps to infer biological meaning and functions affected in LGG and HGG models. In our study, the EMT pathway was found to be significantly upregulated during MT/HMP. *Neuropilin-1 (NRP1)* was detected among top statistically significant DEGs to be highly expressed in HGG vs LGG model. NRP1 acts as a co-receptor for the class 3 semaphorins (SEMA3) and vascular endothelial growth factor (VEGF) [38–40]. Recent studies revealed that NRP1 can actively bind several other growth factors, such as transforming growth factor β1 (TGF-β1) and its receptors, hepatocyte growth factor (HGF) and its receptor cMet, platelet derived growth factor (PDGF) and its receptors, fibroblast growth factors (FGFs), and integrins, which are relevant to angiogenesis, cell proliferation, survival, and wound healing [41, 42]. NRP1 depletion attenuated proliferation and migration ability of cancer cells in vitro and in vivo in multiple cancer models [43, 44]. Overall, studies suggested that in cancer, increased expression of NRP1 has been linked to a poor prognosis and low responsiveness and resistance to radiotherapy and chemotherapy in various cancer types through facilitating aberrant growth factor signaling during EMT-associated drug resistance and metastasis [42, 45]. Another gene found among top significant DEGs between LGG and HGG was *periostin (POSTN*), which interacts with multiple integrins to coordinate a variety of cellular processes, including EMT and cell migration. Several studies report its important role in tumor invasion and resistance to therapy in glioma, breast, lung cancers, renal cell carcinoma [46–49]. Additionally, *ADNP2, PCDH11X,* and *MGST1* detected to be highly expressed in the transformed HGG model have been associated with EMT and treatment resistance in various cancer types [50–53]. Collectively, EMT pathway appears to be an important component driving MT with the development of HMP in IDH-mutant glioma. On the other hand, secreted modular calcium-binding protein 1 (*SMOC1*) was found to be decreased in HGG vs LGG comparison. It appears to be consistent with the report by Wang et al. showing that SMOC1 expression is increased in LGG and significantly correlates with better survival of LGG [54]. In addition, we checked the mRNA expression of DEGs found in our cell models using the TCGA_GBMLGG dataset within GlioVis (http://gliovis.bioinfo.cnio.es), a publicly available web-based portal of brain tumor samples [55]. Upregulation *of NRP1, POSTN, ADNP2* and downregulation of *SMOC1, NOTCH1,* and *BCAN* in the grade 2 vs grade 4 sample comparison were consistent with our findings in LGG and HGG cell models (Suppl. Fig. S6). Given that the models established in this study are representative of the matched tumor samples before and after MT with the development of HMP, it is possible that different sets of DEGs would be present when compared to the matched tumors without the development of HMP. Additional studies analyzing matched tumors of MT without HMP would bring new insights and expand our understanding on the role of HMP during MT.

Additionally, reflecting on the study by Bai et al. [14], we acknowledge that the pathway analysis using fixed tumor tissues may better represent the multicellular complexity and heterogeneity of the primary bulk samples, when compared to monotypic cancer cell populations used in cell models. Despite this limitation, the developed 3D cell lines confer a significant advantage over the fixed single-use tissue analysis as they can be utilized under experimental conditions to further elucidate contributing factors and the biology of the MT process at the cellular level, such as the metabolic reprogramming.

Metabolic rewiring is recognized as a key adaptation for cell survival in IDH-mutant tumors, similar to how genetic and epigenetic changes contribute to tumorigenesis [30, 31, 56]. However, the specific metabolic changes that occur during MT, particularly at the cellular level, are still not well-understood [57, 58]. To address this, we performed metabolic profiling on our established paired model system to explore metabolites within major metabolic pathways. The analysis revealed several prominent metabolic differences between IDH-wt and LGG/HGG IDH-mutant cell lines including accumulation of 2-HG, alterations in the TCA cycle, and suppression of fatty acid synthesis. These findings are consistent with the known effects of IDH mutation on the TCA cycle, where mutant IDH leads to the accumulation of the oncometabolite 2-HG from alpha-ketoglutarate (α-KG) and disrupts normal TCA cycle function [30]. Importantly, when we directly compared LGG and HGG metabolite levels normalized to cell number, we observed elevated glutamine and 2-HG at the cellular level. This aligns with the previous study reporting increased 2-HG levels in higher grade clinical tumor samples compared to lower grade tumors [31], however, our study indicates that the overall increase in 2-HG during MT is due to the changes in the intrinsic metabolism of the tumors and not the increased cellularity which also occurs with MT. In addition, relatively increased acetyl-CoA could be sourced from FAO contributing to elevated citrate to boost TCA cycle and stimulate fatty acid synthesis in 403H.

Glutaminolysis, the conversion of glutamine to glutamate by glutaminase (GLS), serves as a key source for replenishing the TCA cycle intermediate α-KG via anaplerosis [59]. Glutamine also provides essential nitrogen for the synthesis of amino acids and nucleotides, which are crucial for rapid cell proliferation. Consequently, glutamine metabolism is linked to tumor cell survival, maintenance, and antioxidative defense through glutathione (GSH) synthesis. Glutamate and GSH levels were lower in the HGG 403H model while an α-KG was higher, suggesting an increased consumption of glutamate for α-KG production to support TCA cycle and increase 2-HG production as well as reduced antioxidant activity during MT from LGG. These data corroborate previous reports demonstrating that glutamine deprivation and GLS gene silencing reduces glioma cell proliferation, with IDH-mutant glioma cells exhibiting particular sensitivity to GLS inhibitors/glutamine antagonists or GLS gene silencing [60, 61]. As increased glutamine appears to be a major contributor to 2-HG elevation during MT, it is likely that as IDH-mutant LGG transforms to HGG, it becomes even more highly dependent on glutamine metabolism as a critical source of energy for cell growth and survival. However, while removing glutamine from the culture media of LGG and HGG tumors reduced cell viability in our models (data not shown), it didn’t produce a significant shift in the metabolic profiles in both models (Fig. 4A) suggesting that availability of exogenous glutamine may not be the only mechanism driving glutamine dependency during MT.

Reports on the role of glycolysis and lactate production in MT have been complex and somewhat contradictory. Expression of LDHA, which catalyzes oxidation of pyruvate to lactate and other genes involved in glycolysis has been reported to be epigenetically silenced in IDH-mutant compared to IDH-wt cells [62], suggesting that IDH-mutant tumors may be less glycolytic and rely more on mitochondrial metabolism. In contrast, the expression of LDHB, which converts primarily lactate to pyruvate through the reverse reaction, was reported to be increased in IDH-mutant vs IDH-wt models [56, 63, 64]. The hypermethylation of glycolytic enzymes can be lost in IDH-mutant cell models of [65] and ex vivo analysis of patients’ samples has generally shown an increase in lactate upon MT and a negative correlation of LDHA expression with patient survival [58].

Steady state lactate concentrations were lower in our HGG IDH-mutant glioma model and steady state lactate concentrations were lower in both LGG and HGG IDH-mutant models than in IDH-wt model (Fig. 4C and Suppl. Fig. S5). This measurement may depend on the specific metabolic context, however. The LDHA expression was markedly higher in HGG 403H compared to LGG 403L, while the expression of LDHB was at a similarly high level in both. Similarly, when the metabolic flux specifically through the pyruvate to lactate conversion was measured by hyperpolarized MRI, HGG 403H converted significantly more pyruvate to lactate (Fig. 4E), suggesting relative depletion of glycolytic intermediates may be limiting lactate production. Interestingly, we also found that the expression of monocarboxylate transporter 2 (MCT2), which has a high affinity for lactate and is responsible to the uptake of lactate into cells [66], was found to be expressed in much higher levels in 403H than 403L cells (Suppl. Fig. S7). Indirectly, these data implies that HGG tumors may also import lactate via the MCT2 transporter from the TME: astrocytes, neurons, and other cells that produce lactate, and use it, and not glucose, as a direct source of energy [67]. It is possible that such interaction with the TME contributed to the invasiveness and aggressiveness of the HGG 403H tumor growth in vivo. Overall, IDH-mutant gliomas rely on lactate/pyruvate and glutamine/glutamate as anaplerotic substances to support TCA cycle activity, facilitate fatty acid metabolism, and increase 2-HG accumulation during MT.

## Conclusions

We present the first report, to our knowledge, of a 3D model of MT with HMP in IDH-mutant glioma by establishing LGG and HGG stable cell lines derived from the same patient before and after MT. Extensive characterization of the cell line models displayed a close resemblance to the parental tumors and revealed EMT and notch signaling as important pathways altered during MT/HMP. Steady-state metabolic profiling and metabolic flux experiments uncovered increased 2-HG and glutamine levels per cell and enhanced fatty acid metabolism and glycolytic capacity as major metabolic factors of MT/HMP.

## Supplementary Information

Supplementary Material 1.

Supplementary Material 2.

## Data Availability

The RNA sequencing and DNA methylation datasets generated in this study are publicly available in Gene Expression Omnibus (GEO) at GSE267193 (RNA seq) and GSE267615 (DNA Methylation).
