# Supplementary material for: A patient-derived cell model for malignant transformation in IDH-mutant glioma
Source: Acta Neuropathol Commun. 2024 Sep 10;12:148. doi: 10.1186/s40478-024-01860-6 (PMC11385154; doi:10.1186/s40478-024-01860-6)
Supplement: Supplementary file 1 — Supplementary Material 1. [file 40478_2024_1860_MOESM1_ESM.pdf]

Supplementary Figures

A patient-derived cell model for malignant transformation in IDH-mutant glioma

Supplementary Figure 1

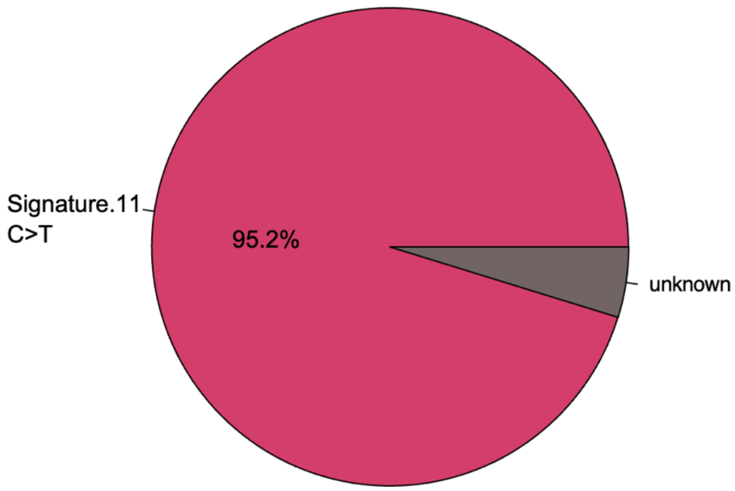

Fig. S1. Mutational signature for 403H tumor sample.

Supplementary Figure 2

A

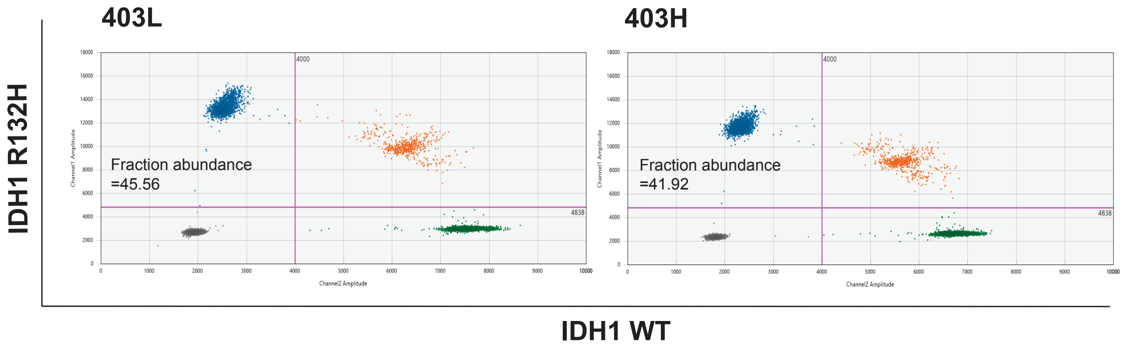

B

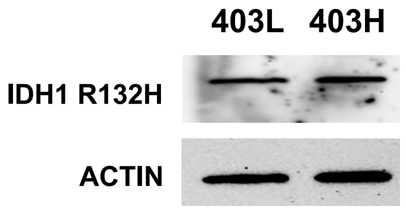

Fig. S2. IDH mutation confirmation in 403L and 403H cells. A. ddPCR result showing the fraction abundance of *IDH1* R132H mutation in both cell lines. **B.** WB of the IDH1 R132H expression in 403L and 403H cells.

Supplementary Figure 3

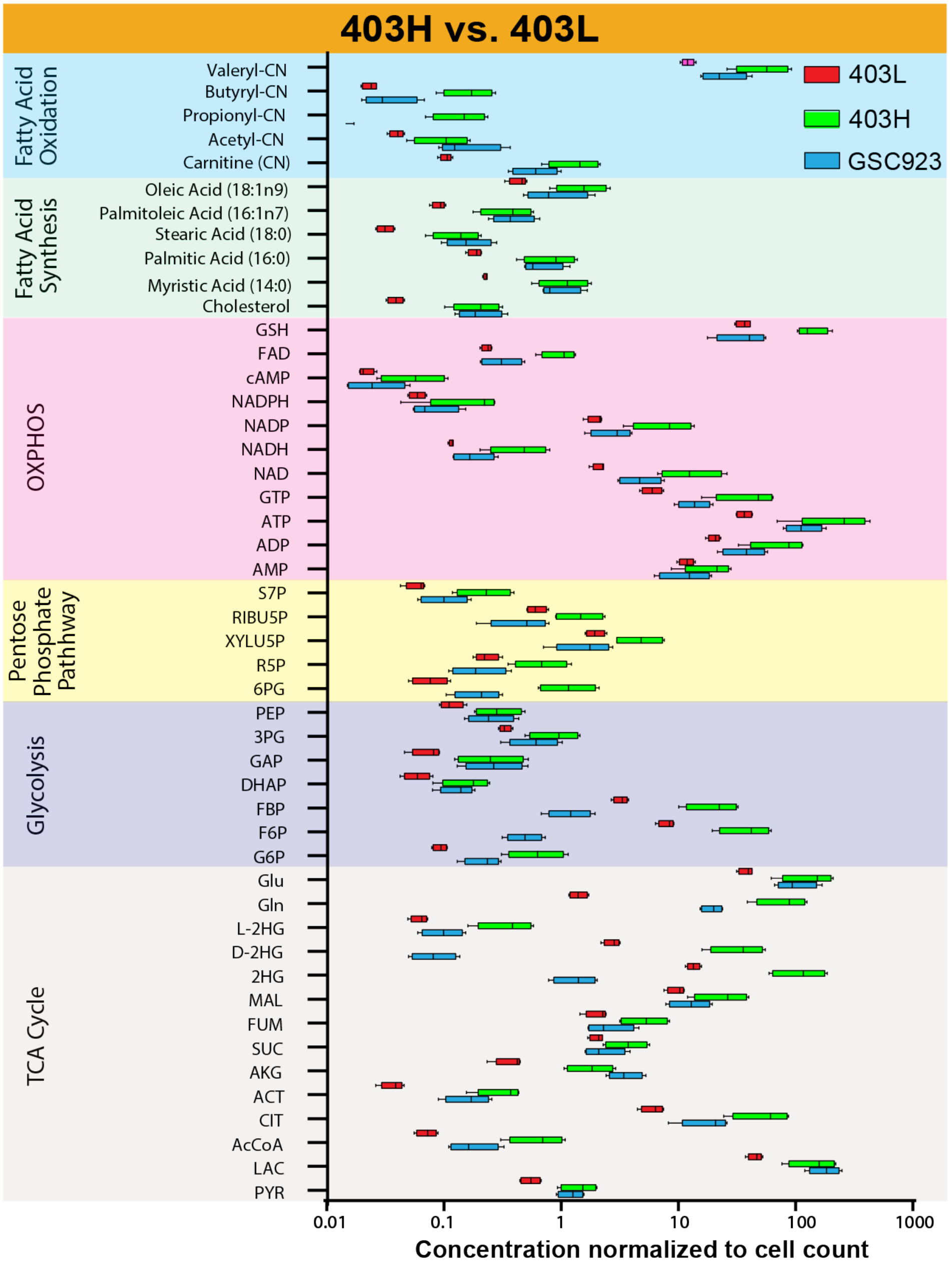

Fig. S3. Metabolite concentrations normalized to cell number for GSC923 (blue), 403L (red), and 403H (green) on a log scale.

Supplementary Figure 4

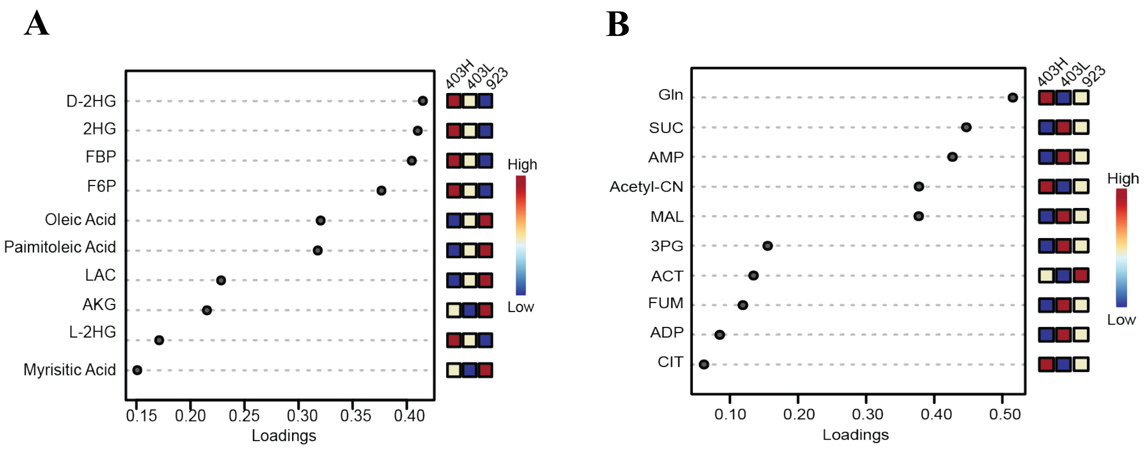

Fig. S4. OPLS-DA plot of the loadings of the first (A) and second components (B) showing the metabolites contributing to the differences between cell lines.

Supplementary Figure 5

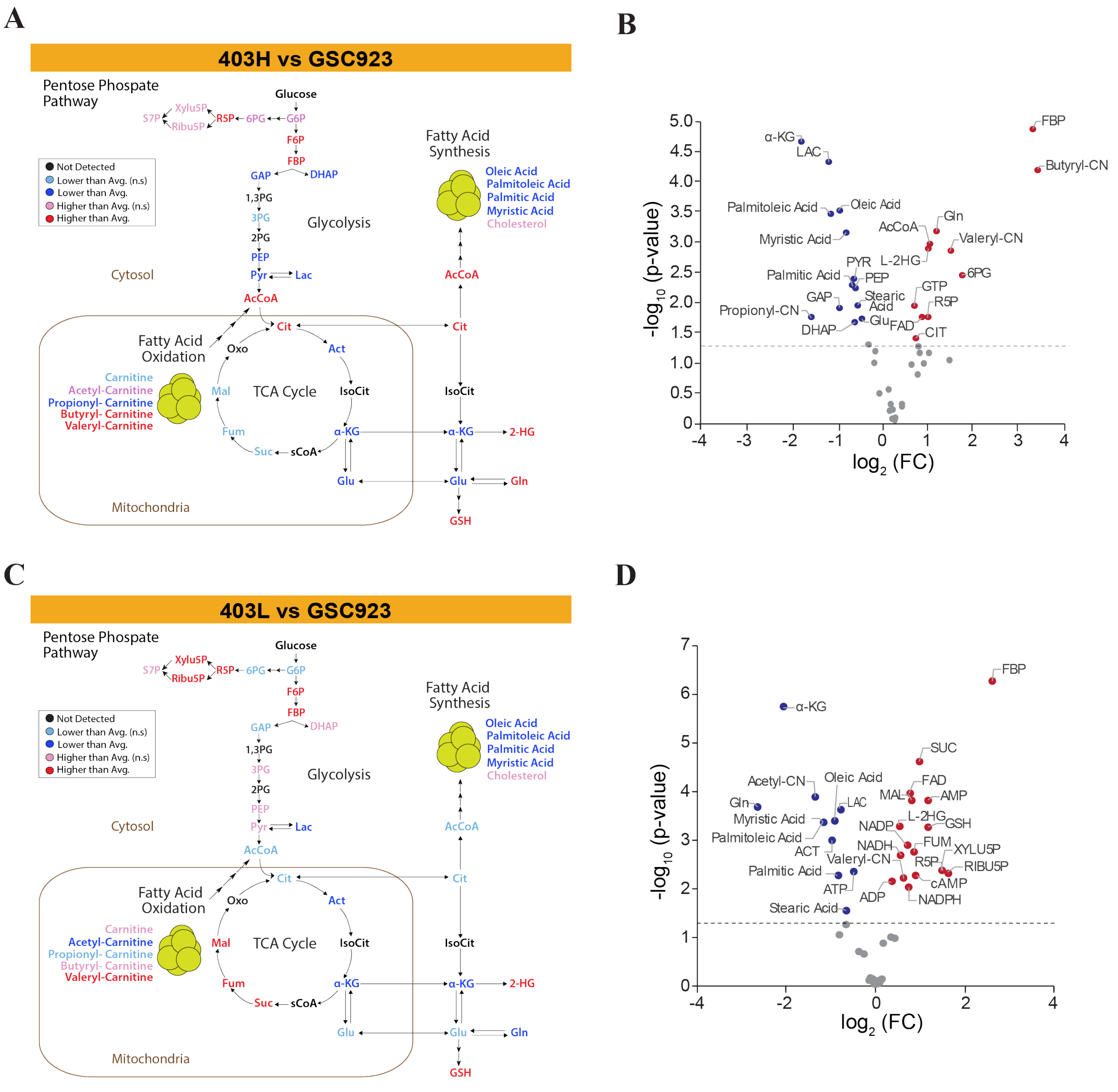

Fig. S5. Comparison of the metabolites in the major metabolite pathways in 403L and 403H vs GSC923. A. Statistically significant metabolites for the 403H vs GS923 comparison plotted on the major metabolite pathways. **B.** Volcano plot for the 403H vs GS923 comparison. **C.** Statistically significant metabolites for the 403L vs GS923 comparison plotted on the major metabolite pathways. **D.** Volcano plot for the 403L vs GS923 comparison.

Supplementary Figure 6

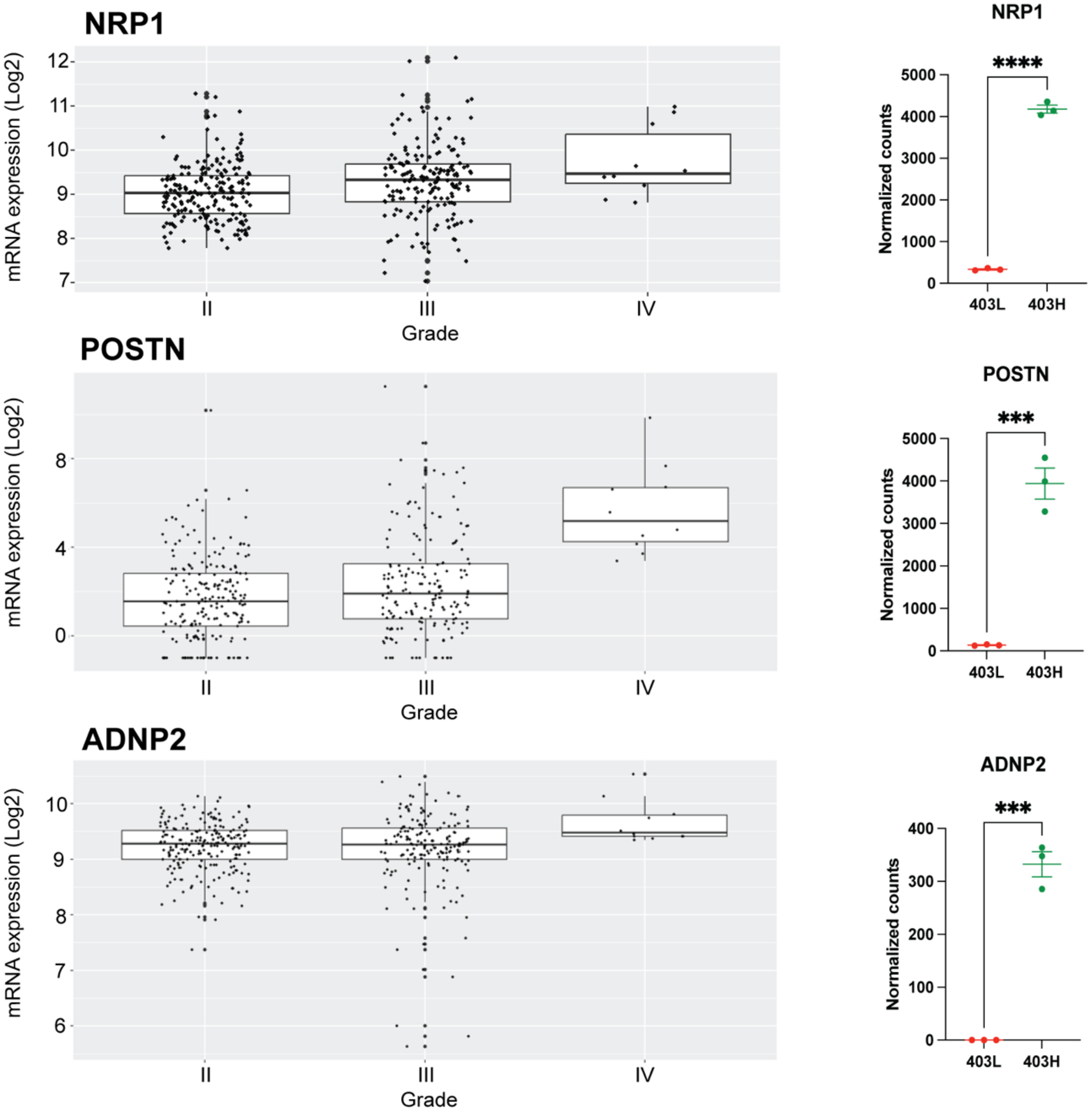

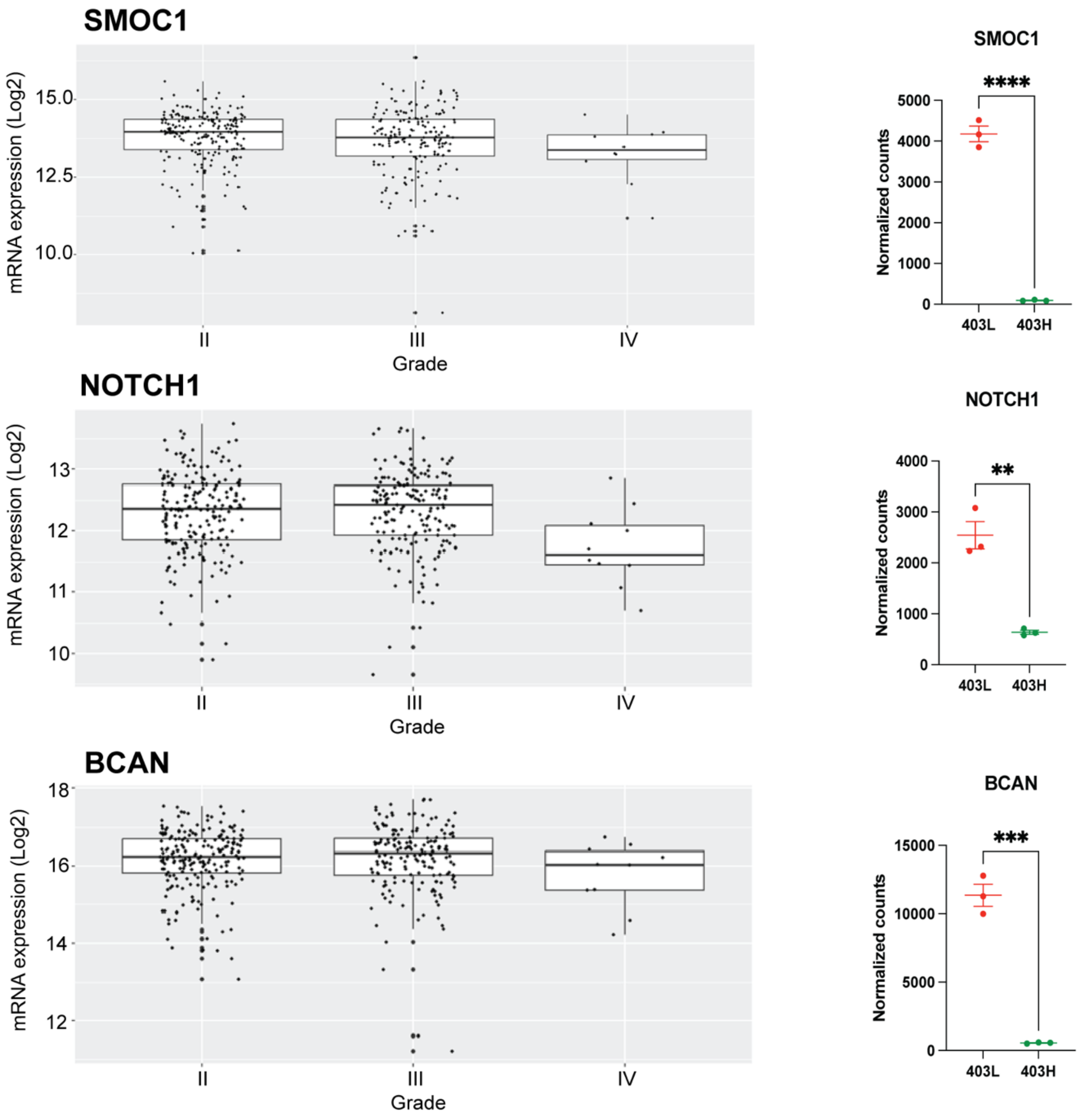

Fig. S6. mRNA expression levels of cell line DEGs analyzed in GlioVis public database portal including only IDH-mutant LGG and HGG TCGA tumor samples along with RNAseq data from 403L and 403H cells.

Supplementary Figure 7

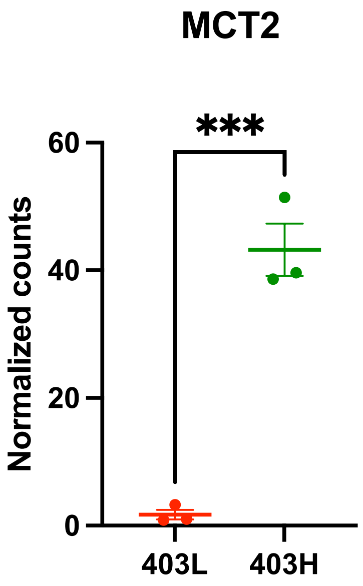

Fig. S7. Normalized counts for MCT2 in 403L and 403H cells based on the RNAseq data.
