# Supplementary material for: A patient-derived cell model for malignant transformation in IDH-mutant glioma
Source: Acta Neuropathol Commun. 2024 Sep 10;12:148. doi: 10.1186/s40478-024-01860-6 (PMC11385154; doi:10.1186/s40478-024-01860-6)
Supplement: Supplementary file 2 — Supplementary Material 2. [file 40478_2024_1860_MOESM2_ESM.docx]

**Supplementary Table S1**. Tier 1 somatic mutations in tumor tissues from resections #1, #2 and #3.

| **Resection #1** | | | **Resection #2** | | | **Resection #3** | | |
| --- | --- | --- | --- | --- | --- | --- | --- | --- |
| Gene | AAChange | VAF | Gene | AAChange | VAF | Gene | AAChange | VAF |
| TP53 | p.Arg273His | 0.769 | TP53 | p.Arg273His | 0.739 | TP53 | p.Arg273His | 0.928 |
| IDH1 | p.Arg132His | 0.371 | IDH1 | p.Arg132His | 0.385 | IDH1 | p.Arg132His | 0.49 |
| ATRX | p.Gln883ArgfsTer13 | 0.576 | ATRX | p.Gln883ArgfsTer13 | 0.468 | ATRX | p.Gln883ArgfsTer13 | 0.578 |
| SETD2 | p.Leu1646HisfsTer20 | 0.103 | SETD2 | p.Leu1646HisfsTer20 | 0.26 | SETD2 | p.Leu1646HisfsTer20 | 0.308 |
| ATM | p.Ser421Ter | 0.11 |  |  |  | CHD4 | p.Trp654Ter | 0.201 |
| PIK3R2 | p.Glu189Ala | 0.176 |  |  |  | PIK3CG | p.Arg1021His | 0.333 |
| IGF2 | p.Thr163Pro | 0.21 |  |  |  | NTRK2 | p.Pro530Ser | 0.401 |
| BCL11A | p.His524Pro | 0.11 |  |  |  | KMT2D | p.Pro2930Leu | 0.468 |
| ETV5 | p.Ala175Pro | 0.188 |  |  |  | RB1 | p.Tyr651Ter | 0.386 |
| TSC1 | p.Glu876ArgfsTer28 | 0.176 |  |  |  | EPHA3 | p.Leu901Phe | 0.434 |
| TSC2 | Splice variant | 0.113 |  |  |  | MSH2 | p.Gln158Ter | 0.379 |
| STAG2 | Splice variant | 0.154 |  |  |  | SETD2 | p.Trp2417Ter | 0.441 |
|  |  |  |  |  |  | TSC1 | Spice variant | 0.484 |

**Annotation**: Tier 1 somatic variants are defined as those occurring in protein-coding regions on hotspot codons, or, if on non-hotspot codons, they must meet at least one of the following criteria: reported as a somatic change in five or more individual tumors, identified as a loss of function in a tumor suppressor gene within the Cancer Gene Census, or recognized as a loss of function by a known mechanism in a non-tumor suppressor gene within the Cancer Gene Census [1].

**References**

1. Roper, N., et al., *Clonal Evolution and Heterogeneity of Osimertinib Acquired Resistance Mechanisms in EGFR Mutant Lung Cancer.* Cell Rep Med, 2020. **1**(1).
